# Role of Serum Creatinine Phosphokinase in Outcome Prediction of Intoxicated Patients; a Brief Report 

**Published:** 2017-03-04

**Authors:** Bita Dadpour, Shahrad Tajoddini, Elham Shaarbaf Eidgahi, Mohsen Shokouhizadeh, Azam Shafahi

**Affiliations:** 1 Medical Toxicology Research Centre, School of Medicine, Mashhad University of Medical Sciences, Mashhad, Iran.; 2 Neuroscience Research Center, Institute of Neuropharmacology, Kerman University of Medical Sciences, Kerman, Iran.; 3 Department of Biostatistics, Faculty of Paramedical Sciences, Shahid Beheshti University of Medical Sciences, Tehran, Iran.; 4 Cardiac Anesthesia Research Centre, Mashhad University of Medical Sciences, Mashhad, Iran.; 5 Addiction Research Centre, Faculty of Medicine, Mashhad University of Medical Sciences, Mashhad, Iran.

**Keywords:** Poisoning, creatine kinase, rhabdomyolysis, emergency service, hospital

## Abstract

**Introduction:**

Several mechanisms were introduced as causes of serum creatinine phosphokinase (CPK) raise in intoxicated patients. This study aimed to assess the relationship between serum CPK level in the first 24 hours and baseline characteristics as well as outcomes of these patients.

**Methods:**

This one year retrospective cross-sectional study was conducted on all intoxicated patients, who were admitted to a referral toxicology center, Northwest of Iran, stayed for at least 24 hours and had serum CPK level more than 500 IU/L in the first 24 hours of admission. The relationship between serum CPK level and some baseline and outcome variables were studied using SPSS version 21.

**Results:**

413 patients with the mean age of 34.52 ± 15.24 years were studied (78.7% male). The mean CPK level at the time of presentation to ED was 3702.85 ± 6375.29 IU/L. There was not any significant relationship between presenting CPK level and type of poisoning (p = 0.258), sex (p = 0.587), and age (p = 0.817). The area under the ROC curve of CPK in prediction of need for dialysis, need for intensive care unit (ICU) admission, and mortality was 0.67 (95% CI: 0.57 – 0.77), 0.60 (95% CI: 0.52 – 0.69), and 0.60 (95% CI: 0.51 – 0.68), respectively.

**Conclusion:**

Based on the finding of present study, there was no significant association between serum CPK level in the first 24 hours and age, sex, and type of poisoning of intoxicated patients and it had poor accuracy in prediction of their need to do dialysis, need for ICU admission, and mortality.

## Introduction

Poisoning has been known as a major cause of emergency department (ED) admission in developing countries and is responsible for a considerable rate of morbidity and mortality (1, 2). It could be followed by various complications such as rhabdomyolysis (3-5). There are some possible risk factors for inducing rhabdomyolysis in this setting and several mechanisms were introduced as causes of serum CPK raise in intoxicated patients. 

Toxic level of many drugs in blood such as antidepressants, isoniazid, antipsychotics, statins, fibrates, and even antihistamines are introduced as predisposing factors of rhabdomyolysis (6). Illegal substances could also lead to muscle distress following overdose or withdrawal (7-9). Serotonergic syndrome and neuroleptic malignant syndrome may be associated with excessive muscle activity or rigidity followed by a rise in serum CPK level (6, 10). Ataxia, seizure and loss of consciousness and further possible prolonged immobility following acute intoxication by many agents put individuals at risk of rhabdomyolysis (6, 10). 

Eizadi-Mood et al. showed that CPK > 10000 IU/L is associated with a higher rate of complication and may be an acceptable predictor of poisoned patients’ outcome (6). Serum CPK level significantly correlates with severity of clinical presentation following organophosphorus poisonings (11). 

This study aimed to assess the relationship between serum CPK level in the first 24 hours and baseline characteristics as well as outcome of intoxicated patients.

## Methods

***Study design and setting***

This retrospective cross-sectional study was conducted on all intoxicated patients presenting to toxicology department of Imam Reza Hospital, Mashhad, Iran, during one year. 

The study followed all recommendations of Mashhad University of Medial Sciences Ethics Committee for descriptive studies.

***Participants***

All intoxicated patients who were admitted to the mentioned toxicology department, stayed for at least 24 hours and had serum CPK level more than 500 IU/L were included, using census sampling. This hospital is a big referral toxicology center in Northwest of Iran.

It is expected that CPK begins to rise 2-12 hours after an acute muscle stress and it will reach its peak plasma level 24 to 36 hours later (12). No confirmed serum CPK level cut off is determined to describe rhabdomyolysis (13). While some studies considered serum CPK level more than 500 IU/L as rhabdomyolysis, others was defined it as a CPK rise more than 5 and 10 times of upper normal limit (12-14).

Patients with history of trauma, infection, myocardial infarction, and electrolytic disturbances were excluded. No sex or age limitation was considered in this study. 

***Data gathering***

A pre-designed checklist that consisted of demographic variables (age, sex), type of poisoning, length of hospital stay, presenting level of consciousness, history of seizure, CPK level in the first 24 hours of presentation to ED, as well as outcomes (need to do dialysis, need for intensive care unit (ICU) admission, and mortality) was used for collection of data. A trained medical student was responsible for reviewing the patients’ medical profiles and data gathering.

***Statistical analysis***

Data were analyzed using SPSS version 21. Findings were reported as mean ± standard deviation or frequency and percentage. Relationship between serum CPK level in the first 24 hours and baseline variables as well as outcome were assessed using chi square or Fisher’s exact tests and area under the receiver operating characteristic (ROC) curve of CPK in prediction of need to do dialysis, need for ICU admission, and mortality were measured with 95% confidence interval (CI).

P < 0.05 was considered significant. 

## Results

413 patients with the mean age of 34.52 ± 15.24 (2 – 90) years were studied (78.7% male). Table 1 shows the baseline characteristics of participants. Most of the patients were in the 20 – 40 years age group (59.6%), and were presented to ED following neuropsychiatric drugs intoxication. The mean duration of hospitalization in this series was 4.30 ± 5.64 (1 – 43) days. 

The mean CPK level during the first 24 hours of presentation to ED was 3702.85 ± 6375.29 (501 – 51220) IU/L. There was not any significant association between serum CPK level in the first 24 hours of presentation and type of poisoning (p = 0.258), sex (p = 0.587), age (p = 0.817), need for ICU admission (p = 0.474), and mortality (p = 0.982). Out of the 24 patients who underwent hemodialysis, 19 (79.2%) had serum CPK level < 10000 (p = 0.002, Pearson's R = - 0.12). The area under the ROC curve of CPK in the first 24 hours of admission for prediction of need to do dialysis, need for ICU admission, and mortality was 0.67 (95% CI: 0.57 – 0.77), 0.60 (95% CI: 0.52 – 0.69), and 0.60 (95% CI: 0.51 – 0.68), respectively. 

**Table 1:** The baseline characteristics of participants

**Variable**	**Number (%)**
**Age (year)**	
< 20	41 (9.9)
20 - 40	246 (59.6)
40 - 60	94 (22.8)
≥ 60	32 (7.7)
**Sex**	
Male	325 (78.7)
female	88 (21.3)
**Type of poisoning**	
Neuropsychiatric drugs	206 (52.0)
Alcohol	133 (33.6)
Carbone monoxide	13 (3.3)
Opium	9 (2.3)
Others	35 (8.8)
**Loss of consciousness**	
Yes	53 (12.8)
No	360 (87.2)
**Seizure**	
Yes	8 (1.9)
No	405 (98.1)
**Dialysis**	
Yes	24 (5.8)
No	389 (94.2)
**ICU admission**	
Yes	45 (10.9)
No	367 (88.9)
**Mortality**	
Yes	38 (9.2)
No	375 (90.8)
**CPK level (** **IU/L)**	
500 - 5000	329 (79.7)
5000 - 10000	55 (13.3)
10000- 15000	10 (2.4)
15000- 20000	4 (1.0)
≥ 20000	15 (3.6)

## Discussion

Based on the findings, there was no significant relation between serum CPK level in the first 24 hours and age, sex, and type of poisoning and it had poor accuracy in prediction of need to do dialysis, need for ICU admission, and mortality of intoxicated patients. 

The most frequent type of poisoning in this study was neuropsychiatric drugs followed by alcohol. This result is similar to the results of Eizadi et al. to some extent, but opioids were the most frequent diagnosis in two other similar studies (6, 15, 16). 

Eizadi-Mood et al. in a prospective study concluded that serum CPK level might be helpful in prediction of poisoned patients' outcome. They reported that higher levels of CPK were followed by increased risk of complications and also death (6). In another study that was retrospectively performed on 114 poisoned patients with rhabdomyolysis, the authors found a significant correlation between serum CPK level and creatinine values, and serum creatinine level, in turn, had a significant relationship with death (15). 

Shadnia et al. reviewing the management of 316 patients with valproate intoxication showed the significant correlation of serum CPK level and outcome in univariate analysis, which was omitted in multivariate analysis (17). 

In contrast, findings of the current study showed that accuracy of serum CPK level in prediction of need for dialysis, need for ICU admission, and mortality is poor. Based on the findings, about 80% of dialyzed patients in this series were among the patients with serum CPK level < 10000 IU/L. 

Since there is no consensus regarding the best cut off point of CPK in prediction of outcome, we used area under the ROC curve for presentation of its accuracy in this regard.

It seems that, even though the relationship between serum CPK level and intoxicated patients’ outcomes was reported in some studies, it needs to be confirmed in other studies with larger sample size and multivariate analysis models, considering other probable predictors of outcome. 

***Limitation***

Doing the study in a retrospective fashion, not considering other probable predictive factors of outcome, and not considering the time lag between toxic agent consumption and presenting to ED were among the most important limitations of the study, which distort the generalizability of the findings. 

## Conclusion:

Based on the finding of present study, there was no significant association between serum CPK level in the first 24 hours and age, sex, and type of poisoning in intoxicated patients and it had poor accuracy in prediction of their need to do dialysis, need for ICU admission, and mortality. 
